# Growth hormone reverses dyslipidemia in adult offspring after maternal undernutrition

**DOI:** 10.1038/s41598-017-05045-1

**Published:** 2017-07-20

**Authors:** Wei-fen Zhu, Sheng-jie Tang, Zheng Shen, Ying-min Wang, Li Liang

**Affiliations:** 1 Department of Pediatrics, The First Affiliated Hospital, College of Medicine, Zhejiang University, Hangzhou, China; 2 Department of Endocrinology, Sir Run Run Shaw Hospital, College of Medicine, Zhejiang University, Hangzhou, China; 3 Department of Central Laboratory, Children’s Hospital of Zhejiang University School of Medicine, Hangzhou, China; 4 Department of Endocrinology, Tongde Hospital of Zhejiang Province, Hangzhou, China

## Abstract

The abnormal intrauterine milieu of fetal growth retardation could lead to dyslipidemia in adulthood. Studies have shown that growth hormone (GH) therapy in small for gestational age (SGA) children would be beneficial for metabolic parameters. Here we investigated whether GH treatment introduced at adolescent period in SGA could reverse dyslipidemia during later life. SGA rat model was established by using semi-starvation treatment during the whole pregnancy. SGA or appropriate for gestational age (AGA) offspring were assigned to receive GH or normal saline (NS). Once-daily subcutaneous injections of GH were administered between 21–35 days of age. In adulthood, as compared to AGA, SGA showed: (1) decreased body weight and length; (2) increased serum triglycerides; (3) down-regulated hepatic AMPK-α1 but up-regulated SREBP-1c and ACC-1; (4) a significant reduction in histone H3 acetylation at the promoter of AMPK-α1. Exogenous GH administration led to a restoration of body weight and length and normalized serum triglycerides by reversing expression of AMPK-α1 and its targeted genes SREBP-1c and ACC-1, through increasing H3 acetylation at the promoter of AMPK-α1 in SGA in adult period. These results demonstrated positive effects on lipid metabolism by a short treatment course of GH in SGA adult period.

## Introduction

Small for gestation age (SGA) is a common complication of pregnancy and a significant cause of perinatal morbidity and mortality^1^. With the increased use of assisted reproductive techniques and increasing number of advanced maternal age, the overall incidence of SGA is as high as 8.77% in China^2^. Many lines of clinical evidence indicate that adverse conditions during critical time windows of development can alter physiological processes leading to metabolic diseases, such as central obesity, dyslipidemia and cardiovascular diseases^3, 4^. We have previously demonstrated that maternal undernutrition during pregnancy alters serum lipid profiles not only in early adolescence, but also in adult periods^5^. Thus, as a serious public health problem, it is imperative to study the outcomes of the individuals that develop as SGA babies, helping to design strategies to halt the current epidemic in metabolic diseases.

AMPK, known to be a major regulator in energy homeostasis, is a heterotrimeric enzyme complex consisting of a catalytic subunit (α) and two regulatory subunits (β and γ). Two isoforms are known for catalytic subunit (α1, α2) and the expression of them in liver tissue is approximately equal^6^. AMPK’s role in lipid metabolism has been highlighted in recent years. AMPK could inhibit both cholesterol and fatty acid biosynthesis by inactivating acetyl CoA carboxylase (ACC) and 3-hydroxy-3-methylglutaryl CoA reductase in the form of short regulation, and regulate hepatic lipogenic gene expression by inhibiting SREBP-1c, peroxisome proliferator-activated receptors or carbohydrate responsive element-binding protein in the form of long regulation^7, 8^. Previous study suggested that undernutrition stress in utero might induce postnatal suppression of hepatic AMPK activities and elevation of SREBP-1c expression, resulting in increased lipogenesis, decreased lipolysis and increased fat stores^9^. It is known that AMPK could be activated by numbers of physiologic and pathophysiologic signals such as exercise, hormones, and hypoxia^10^. In addition, activating AMPK with metformin or natural compounds could prevent the development of hepatic sclerosis, hyperlipidemia, and accelerated atherosclerosis^11, 12^. In Yang’s study, they generated a new transgenic mouse model expressing constitutively active AMPK-alpha1 in liver and demonstrated that chronic activation of AMPK-α1 decreases expression of SREBP-1c and its target genes, which results in reduced fat storage^13^. Knowles *et al*. showed that the constitutively active AMPK-a1 transgenic mice at 10–12 weeks of age exhibited normal hepatic triglyceride content as compared to wild type mice by inhibiting 3-hydroxy-3-methylglutaryl coenzyme A reductase and acetyl CoA carboxylase^14^. Thus, we predicted that inhibiting lipogenesis via activating the AMPK-a1/SREBP-1c pathway might contribute to the protective activity against dyslipidemia in SGA rats.

Growth hormone (GH) is the main regulator of postnatal growth, which has been considered as therapeutic agent to treat growth failure in SGA who miss catch up until the age of two^15^. Many clinical studies have shown that GH therapy in SGA would not only be effective for adult stature, but also be beneficial for various metabolic parameters, such as lipid profiles, systolic and diastolic blood pressure^16, 17^. In recent years, influences of GH on lipid metabolism got more and more attentions. In Qin’s study, they demonstrated that chronic exogenous GH administration (via gene therapy) could improve body composition, ameliorate serum lipid profiles and decrease oxidative stress by activating the hepatic adiponectin-SIRT1-AMPK and PPARα-AMPK signaling systems^18^. Olsson showed that the expression of SREBP-1 was decreased in bovine GH-transgenic mice livers, what’s more, they also noted a decreased expression of genes regulated by SREBP-1, the HDL receptor scavenger receptor class B type I, ATP-citratelyase, glycerol-3-phosphate acyltransferase, fatty acid synthetase, stearoyl-CoA desaturase, and spot 14^19^. Utilising our well established model of maternal undernutrition to induce developmental programming, we aimed to investigate whether GH treatment introduced at adolescent period could reverse the development of dyslipidemia during later life by activating AMPK-SREBP pathway in rat model.

## Results

### GH treatment reverses the growth retardation in SGA

**Figure 1:**
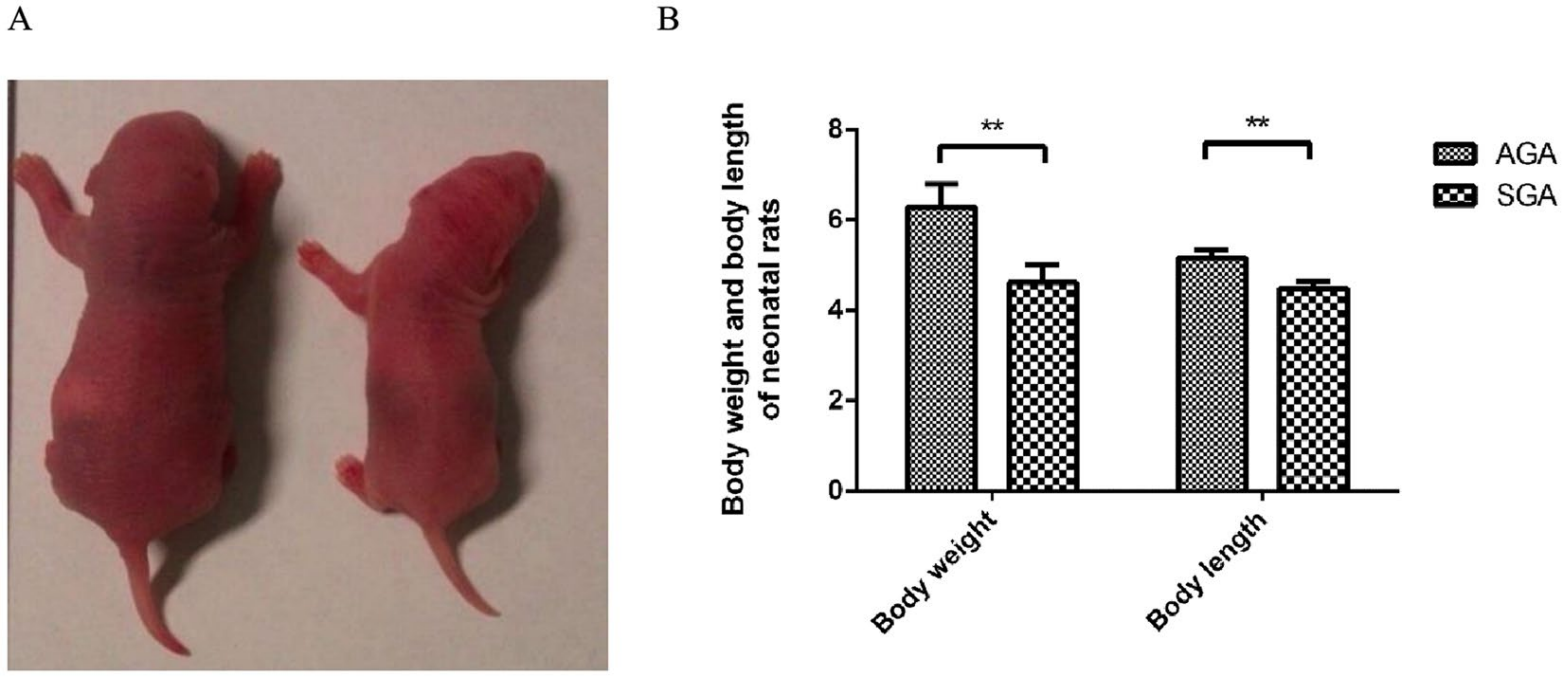
The characteristics of newborn rat pups. The photograph (**A**) and body weight and body length (**B**) of newborn rat pups. Statistical differences between the groups are given as follows: **p < 0.01: AGA vs SGA. n = 16 per experimental group.

Maternal undernutrition during pregnancy resulted in fetal growth retardation, the SGA neonatal offspring is characterized by significantly lower body weight and body length at parturition (all *P* < 0.01, Fig. 1). From 1 to 21 days of age, SGA rats exhibited catch-up growth, however, the body weight and body length of which remained significantly lower than AGA rats at 21 days of age (*P* < 0.05 and *P* < 0.01). GH treatment led to a restoration of body weight and body length in SGA rats at 70 days of age, an effect that persisted into adulthood (Table 1).

**Table 1:** The body weight and body length before and after GH or NS treatment.

		AGA + NS	AGA + rhGH	SGA + NS	SGA + rhGH
Body weight, g	Day21	53.18 ± 2.91		49.26 ± 3.25*	
	Day35	147.44 ± 6.25	154.45 ± 8.28	133.08 ± 10.33^&^	146.19 ± 9.15^#^
	Day70	361.83 ± 10.78	369.38 ± 7.65	339.67 ± 14.19^&^	356.32 ± 10.59^#^
Body length, cm	Day21	12.16 ± 0.13		11.28 ± 0.16**	
	Day35	17.58 ± 0.65	17.76 ± 0.85	16.43 ± 0.49^&^	17.23 ± 0.67
	Day70	22.45 ± 1.48	22.94 ± 1.53	20.31 ± 1.41^&^	22.46 ± 1.73^#^

ANOVO: Results are expressed as the mean ± S.E.M. Statistical differences between the groups are given as follows: *p < 0.05: AGA vs SGA; **p < 0.01: AGA vs SGA; ^&^ *p* < 0.05: AGA + NS vs AGA + rhGH; ^#^ *p* < 0.05: SGA + NS vs SGA + rhGH^.^ n = 8 per experimental group.

### GH treatment reverses serum triglycerides

**Figure 2:**
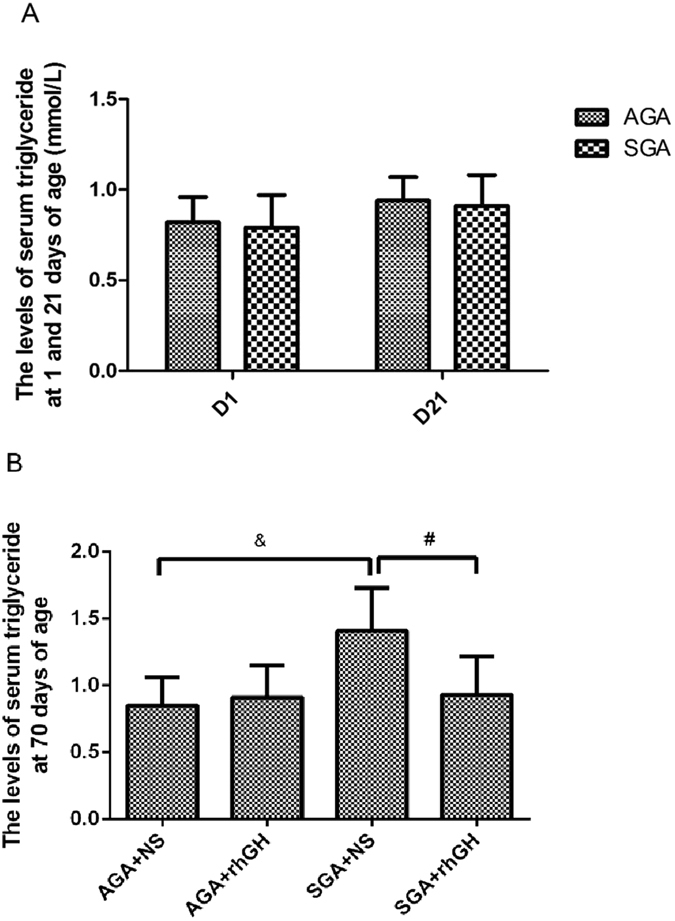
The levels of serum triglyceride before and after GH or NS treatment. Results are expressed as the mean ± S.E.M. Statistical differences between the groups are given as follows: &p < 0.05: AGA + NS vs SGA + NS; #p < 0.05: SGA + NS vs SGA + rhGH. n = 8 per experimental group.

There were no differences between SGA and AGA rats at 1 or 21 days of age (Fig. 2A). Serum triglycerides were significantly reduced following GH-treated rats compared with NS-treated rats (*P* < 0.05) in SGA, but were unaltered with GH or NS treatment in AGA rats, indicating that exogenous GH administration normalized serum triglycerides in SGA rats (Fig. 2B).

### GH treatment reverses protein expression of AMPK-α1/SREBP-1c/ACC-1 pathway

**Figure 3:**
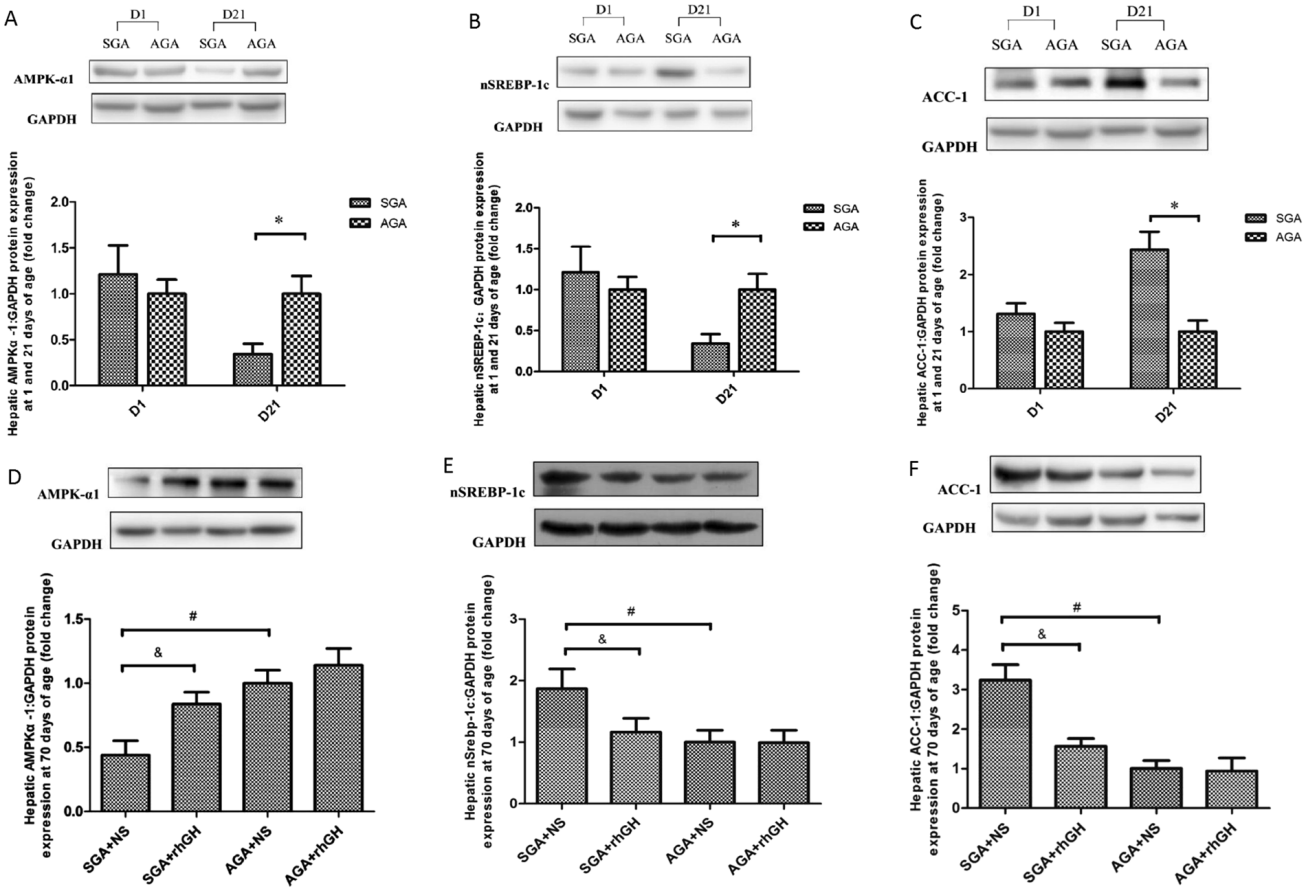
Immunoprecipitation/western blot analyses of hepatic AMPK-α1, nSREBP-1c and ACC-1 protein expression. Immunoblots were quantified using densitometry and normalized to GAPDH protein expression. Results are expressed as fold change compared to the AGA or AGA + NS of same age. Hepatic AMPK-α1 protein expression before (**A**) and after (**D**) GH or NS treatment. Hepatic nSREBP-1c protein expression before (**B**) and after (**E**) GH or NS treatment. Hepatic ACC-1 protein expression before (**C**) and after (**F**) GH or NS treatment. Statistical differences between the groups are given as follows: *p < 0.05: AGA vs SGA; ^#^ *p* < 0.05: AGA + NS vs SGA + NS; ^&^ *p* < 0.05: SGA + NS vs SGA + rhGH. n = 8 per experimental group.

AMPK-α1 is known to be a major regulator of the lipid biosynthetic pathway. We examined the effects of maternal undernutrition on the hepatic AMPK, SREBP-1c and ACC-1 expression in SGA rats. Western blooting analysis showed that maternal undernutrition significantly down-regulated hepatic AMPK-α1 (*P* < 0.05) but up-regulated SREBP-1c and ACC-1 protein levels (*P < *0.05) compared to the AGA rats at 21 days of age (Fig. 3A,B and C). These changes were reversed by GH in adulthood (Fig. 3D,E and F).

### GH treatment reverses mRNA expression of AMPK-α1/SREBP-1c/ACC-1 pathway

**Figure 4:**
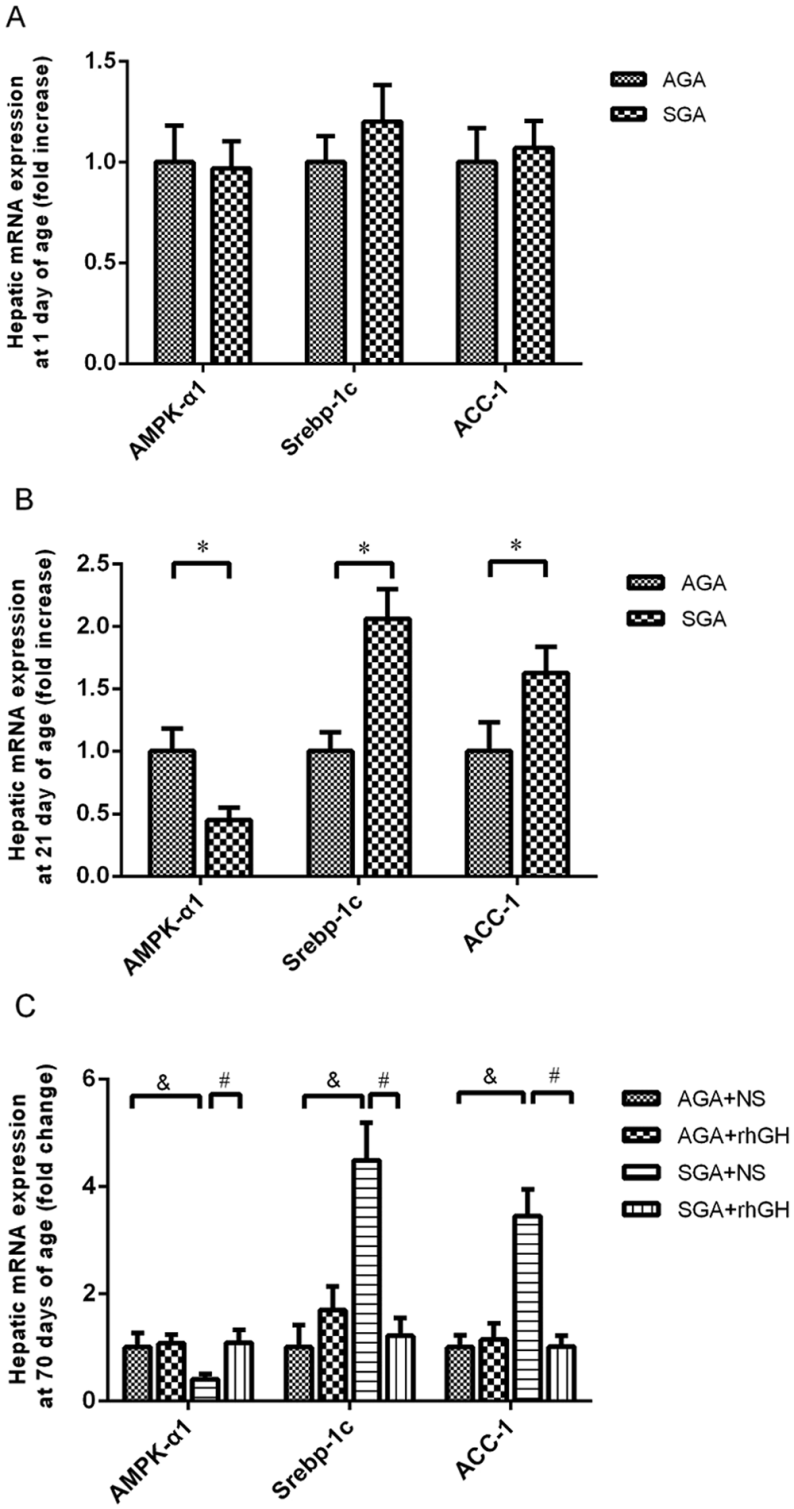
Real-time RT-PCR analysis of hepatic AMPK-α1, nSREBP-1c and ACC-1 mRNA expression. Results are expressed as fold change compared to the AGA or AGA + NS of same age. Hepatic mRNA expression at neonatal (**A**) adolescent (**B**) and adult (**C**) periods. Statistical differences between the groups are given as follows: *p < 0.05: AGA vs SGA; ^&^ *p* < 0.05: AGA + NS vs SGA + NS; ^#^ *p* < 0.05: SGA + NS vs SGA + rhGH. n = 8 per experimental group.

The 21-day-old SGA rats demonstrated a marked decrease in mRNA expression of AMPK-α1 (*P* < 0.05), a massive increase in mRNA expression of SREBP-1c (*P* < 0.01) and ACC-1 (*P* < 0.05) when compared to AGA rats at the same age. Exogenous GH administration significantly increased hepatic AMPK-α1 mRNA expression (*P* < 0.05) and decreased SREBP-1c and ACC-1 mRNA expression (all *P* < 0.05) in the SGA + GH group compared with the SGA + NS group (Shown in Fig. 4).

### GH treatment restores histone acetylation at the AMPK-α1 promoter

**Figure 5:**
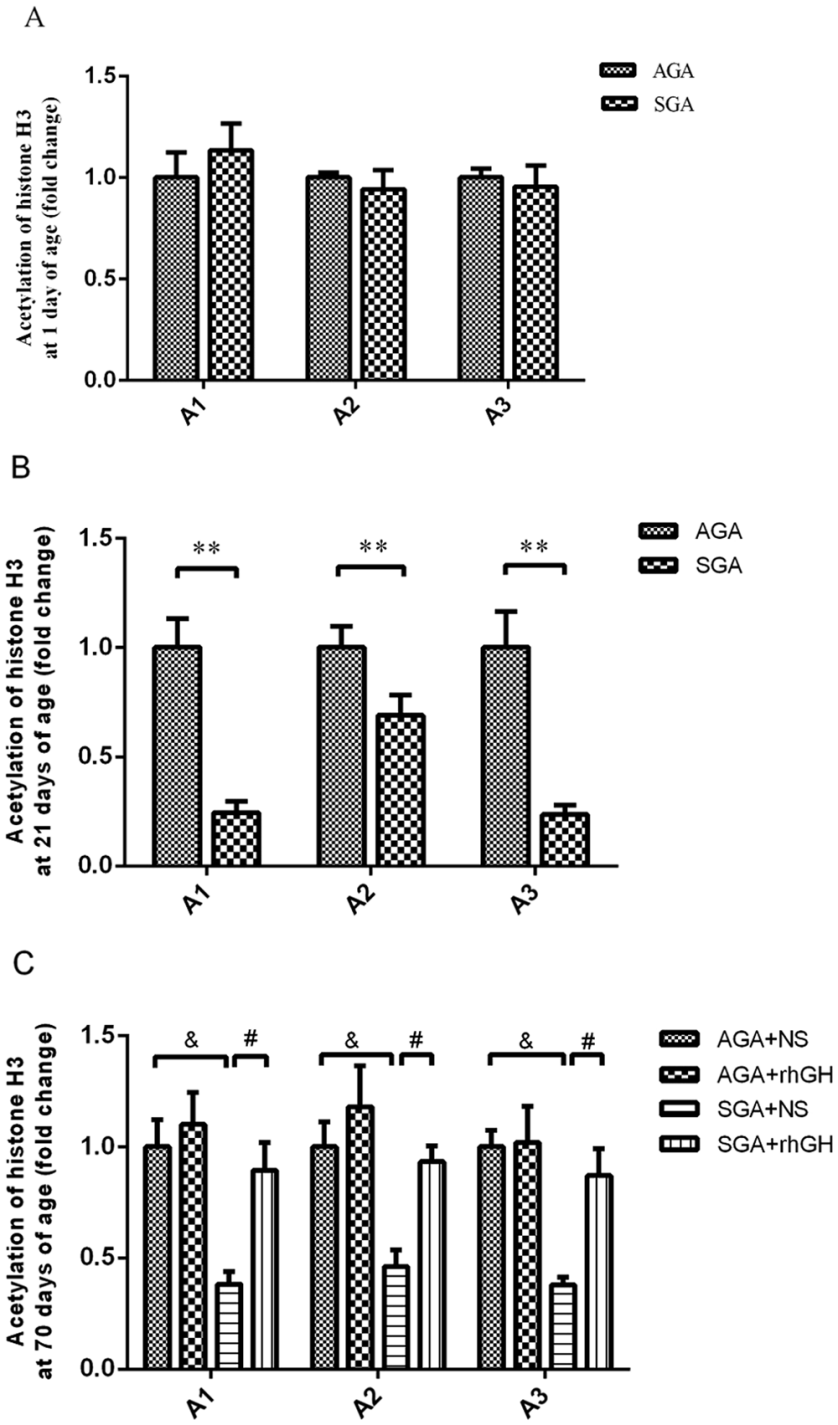
ChIP-PCR analysis of H3 acetylation at the promoter of AMPK-α1 with antibody to histone H3. Acetylation of histone H3 at neonatal (**A**) adolescent (**B**) and adult (**C**) periods. Quantification was performed using real-time PCR with primers specific to the proposed AMPK-α1 element sites (A1: −449 to −640; A2: −887 to −1099; A3: −1330 to −1526). Statistical differences between the groups are given as follows: **p < 0.01: AGA vs SGA; ^&^ *p* < 0.05: AGA + NS vs SGA + NS; ^#^ *p* < 0.05: SGA + NS vs SGA + rhGH. n = 6 per experimental group.

Histones modification is the predominant epigenetic phenomena that could control gene expression by modulating the structure of chromatin and the accessibility of regulatory DNA sequences to transcriptional activators and repressors. To determine whether the restoration of AMPK-α1 transcription in SGA GH treated animals was due to increased acetylation of H3 at the proximal promoter of AMPK-α1, we employed ChIP coupled with quantitative PCR. Mainly three sites (A1: −449 to −640; A2: −887 to −1099; A3: −1330 to −1526) along the AMPK-α1 promoter were analyzed (Shown in Fig. 5). At 21 days of age, there were significant reductions in histone H3 acetylation at the promoter of AMPK-α1 in SGA liver tissue compared with AGA rats. Furthermore, GH treatment increased histone H3 acetylation at 70 days of age in SGA rats, but had no significant effect in AGA rats, consistent with changes in expression of AMPK-α1 in response to GH treatment. When the CHIP experiments were carried out with IgG antibody, only a negligible amount of immunoprecipitations were observed, indicating that the proteins of interest protein binding at the AMPK-α1 promoter were successfully measured compared with non-specific protein.

## Discussion

The “fetal origins of adult disease hypothesis” put forward by Barker suggests that fetal adaptation to an adverse intrauterine milieu leads to persistent changes in cellular biology and systemic physiology^20^. To understand the molecular mechanisms, various animal models have been developed to promote intrauterine fetal programming, including maternal undernutrition, uteroplacental insufficiency and maternal glucocortcoid exposure^21^. In our study, SGA model was achieved by prenatal nutrient restriction, which could obtain higher success rates. SGA rats grew faster since birth, by 21 days of age, however, they still remained shorter and lighter than their normal counterparts, failed to fully catch up growth, which were similar with Chakraborty’s clinical study^22^. GH treatments in our study were effective, significantly preventing growth retardation in SGA rats and maintaining it close to normal levels.

Our study observed that the adult SGA rats showed weak ability to handle a lipid load resulting in increased serum TG levels, coincident with Choi’s study^23^. It has been reported that lipid metabolism might be more sensitive to malnutrition during intrauterine development of fetus^24–26^. Here we speculated that hypertriglyceridemia observed in adult SGA rats exposed to maternal undernutrition during pregnancy might be a result of abnormal expression of AMPK-α1/SREBP-1c/ACC-1 pathway. The disturbance of serum triglycerides in SGA rats was not permanent, which could be ameliorated by GH treatment, might through AMPK-α1/SREBP-1c/ACC-1 pathway. As numbers of studies have indicated that in liver tissue GH can interact with its target cell surface receptor and induces tyrosine phosphorylation of GHR and JAK2, consequently activating multiple signaling molecules, including p38 mitogen activated protein kinase (p38-MAPK), PPARα and AMPK, it is likely that this is the mechanism underlying GH induced increased association at AMPK-α1 in SGA liver^27–29^. In Mueller’s study, they showed that GH treatment led to decreased hepatic SREBP-1c expression in wt mice, which might be associated with a transcriptional inhibition by GH-activated STAT5 on SREBP-1c gene promoter^30^. Thus, the decreased expression of SREBP-1c might be not only associated with AMPK-α1 activation, but also related to STAT5 pathway.

Another conclusion that can be extrapolated from this study is that histone modification might be partly responsible for the increased transcription of AMPK-α1 gene. Considering acetylation of histone H3 are known to be hallmarks of chromatin opening^31^, our findings discovered that the AMPK-α1 expression is repressed through histone hypoacetylation at weaning and early adult stage. Combined with our data and many previous studies^32–34^, we believed that epigenetics could mediate the effects of fetal programming long term into adulthood. It has been known for a long time that the epigenetic gene regulation in mammalian development is highly flexible^35^, thus, reversing epigenetic modifications at early postnatal life would be better for preventing adverse hepatic outcomes in SGA rats. In our previous study, we showed that GH treatment had an impact on increasing PDX1 expression probably through epigenetic mechanism which includes upregulated Set7/9 expression and decreased HDAC activity in SGA islet^36^. Chia *et al*. showed that GH could induced a dramatic rise in acetylation of core histones at the IGF-1 promoters through recruiting histone acetyltransferases to the IGF-1 promoters (e.g. p300-associated P/CAF) or interfering the activity of HDACs^37, 38^. Here, we speculated GH treatment during 3–5 weeks of age (at an early age) promoting acetylation of histone H3 in the promoter of AMPK-α1 would be related to the prevention of HDAC activity. Certainly, further studies are needed to explore the possible mechanisms.

Interestingly, GH treatment have different effects in lipid metabolisms in previous studies. Qin *et al*. demonstrated that GH administration via gene therapy (rAAV2/1 vector containing the GH1 gene) reversed the increased hepatic TG in animal models of alcoholic fatty liver disease after 6 weeks of alcohol exposure by reduced hepatic nSREBP-1c protein expression and stimulated hepatic AMPKα and PPARα activity^39^. In Donkin’s study, GH given as daily injections to ovariectomized female rats, mimicking the male secretory pattern of GH, decreased FAS mRNA expression^40^. On the contrary, GH treatment given by means of Alzet osmotic minipumps implanted subcutaneously, mimicking the female secretory pattern of GH, resulted in increases in SREBP-1c and hepatic expression of lipogenic enzymes in hypophysectomized rats^41^. The different endpoints of the study, differences in the animal models, the given pattern of GH, and the dose of GH may in part explain the contrasting findinds.Although GH treatment after weaning had positive effects on lipid metabolism in SGA, there are several limitations to be discussed in our study. Firstly, it is unclear whether the protective effects of GH against hypertriglyceridemia through directly or indirectly through the activation of this signaling pathway. Secondly, it is possible that GH influences signaling pathways other than those described in this study. Finally, although this study offers a good point for the GH therapy in SGA, more studies are needed to confirm the encouraging results in clinical medicine.

## Conclusions

In short, our work demonstrated that maternal undernutrition induced SGA led to increased serum triglyceride in later life, through altering the expression of key factors involved in the regulation of triglyceride metabolism. What’s more, an early GH treatment intervention after weaning might reverse this “in utero imprint” on AMPK-α1/SREBP-1c/ACC-1 pathways through epigenetic regulation.

## Methods

### Animal model

All procedures used in this study were approved and performed in accordance with the guidelines established by the Animal Ethics Committee of the Zhejiang University School of Medicine.

Female Sprague-Dawley rats weighing 250 ± 10 g (mean ± SD) were obtained from SLRC Laboratory Animal (Shanghai, China).The rats were housed in separate cages (located in Laboratory Animal Center Of Zhejiang University) maintained with controlled temperature (23 ± 2 °C), humidity (40–65%) were cohabitated with adult male rats for mating. Conception was confirmed by examination of vaginal smear for the presence of sperm on the day after mating. Rats were provided either an ad libitum diet of standard laboratory chow or a 50% food-restricted diet determined by quantification of normal intake in ad libitum fed rats, from day 1 of pregnancy until parturition. The standard commercial rat diet contained (per 100 g) protein (22.5 g), carbohydrates (57.0 g), fat (3.9 g), cellulose (8.0 g), minerals (1 g), vitamins (mixed, 5.0 g), and water (2.5 g). The pregnant rats delivered spontaneously and after parturition, feeding was ad libitum in both groups. The offspring of normal intake and birth weight between mean ± SD were defined as AGA; the offspring of food-restricted mothers and birth weight less than -2 SD of AGA group were defined as SGA. Little size was adjusted to 8 pups per littler to assure adequate and standardized nutrition until weaning.

### GH treatment

At postnatal day 21, rats were treated with human recombinant GH (rhGH, Jintropin® AQ, GenSci, Changchun, China) in a dose of 5 mg/kg per day in one single intraperitoneal injection at 9.00 am. In vehicle treated rats an equivalent volume of “saline” was injected at the same time. The dose of rhGH treatment were chosen according to the study of Vickers’ study^42^. Four experimental groups were created: AGA rats receiving rhGH treatment (AGA + rhGH, n = 8), AGA rats receiving NS treatment (AGA + NS, n = 8), SGA rats receiving rhGH treatment (SGA + rhGH, n = 8), and SGA rats receiving NS treatment (SGA + NS, n = 8). Body weight and length, food and water intakes of all rats were measured daily throughout the experiment. At postnatal day 35, the body weight and length of SGA + rhGH were similar to AGA + NS, thus we stopped the GH or NS treatment. Rats were fasted overnight and killed by anesthesia with chloral hydrate for study at 1 (neonatal stage), 21 (puverty stage) and 70 (adult stage) days of age. We did not choose the female offspring for study in order to prevent confounding factors related to their estrus cycle and hormone profile.

### Serum TG levels

Serum TG levels were measured using commercial kits (catalog no. F001-2, Nanjing Jiancheng Bioengineering Institute, China) and employing enzymatic assay of GPO-PAP method. Reagents and samples were prepared according to the manufacturer’s instructions, and absorbencies were measured at 500 nm using a spectrophotometer.

### Western-blot

Protein concentrations were determined with Bicinchoninic Acid assay (Beyotime biotechnology, Hangzhou, China). Thirty microgram of liver protein extracts were heated for 7 min at 95 °C and then subjected to 10% SDS-PAGE gels using the Bio-Rad Mini Protean II system, and proteins were electroblotted onto polyvinylidene difluoride membranes (0.2 μm pore size, catalog No. ISEQ00010, Millipore, Billerica, MA, USA). Blocking was carried out with freshly prepared PBS plus 5% nonfat milk. After washing, the membrane was incubated overnight at 4 °C with anti-AMPK-α1 antibodies (dilution 1:1000, catalog no. 1596–1), anti-SREBP-1c antibodies (dilution 1:3000, catalog no. ab3259, Abcam, New Territories, HK) and anti-GPADH antibodies (dilution 1:5000, catalog no. 5174, Cell Signaling Technology, Boston, USA), and subsequently incubated with secondary antibodies conjugated with horseradish peroxidase (Beyotime biotechnology, Hangzhou, China) for 2 h at room temperature. Signals were detected using ECL performed according to the manufacturer’s instructions (Supersignal, Pierce, Rockford, IL, USA).

### RNA isolation and real-time PCR analysis

Total RNA was isolated using the Total RNA Miniprep Kit (catalog no. 74104, Qiagen, Hilden, Germany) and quantified at 260 and 280 nm using a spectrophotometer. Total RNA (1 ug) from was reversely transcribed to cDNA in a total volume of 20 ul, using High-Capacity cDNA Reverse Transcription Kit (Applied Biosystems, Foster City, CA, USA) with a thermal cycler with the following program: 25 °C for 10 min, 37 °C for 120 min, 85 °C for 5 min, and a 4 °C hold. Real-time PCR was performed using 1 ng of cDNA as the template, SYBR Green PCR Master Mix (Applied Biosystems, Foster City, CA, USA), and 4 uM of each forward and reverse primer in the Applied Biosystems StepOnePlus Real-Time PCR System with the following program: initial denaturation at 94 °C for 10 min, 40 cycles consisted of denaturation at 94  °C for 20 s, 60 °C for 1 min for annealing and extension. Relative quantifications of PCR products were based on the ^ΔΔ^ *C*_t_ method. The housekeeping mRNA glyceraldehydes-3-phosphate dehydrogenase (GAPDH) was used as an internal control. Sequencing Primers are as follows: AMPK-α1: forward: 5′-ATTGGATTTCCGAAGTATTGATG-3′, reverse: 5′-CCTGGTCTTGGAGCTACGTCA-3′; SREBP-1c: forward: 5′-CCTGTAGGTCACCGTTTCTT-3′, reverse: 5′-GTTCACAGAATAGTCGGGTCAC-3′; ACC-1: forward: 5′-GGCGACTTACGTTCCTAGTTG-3′, reverse: 5′-AGATGTCGATAAATGCGGTCC-3′; GAPDH: forward: 5′-TGGTCTACATGTTCCAGTATGACT-3′, reverse: 5′-CCATTTGATGTTAGCGGGATCTC-3′.

### Chromatin immunopreciptation

Chromatin immunopreciptation (ChIP) was performed by EZ-CHIP Kit according to the manufacturer’s instructions (catalog no. 17–371, Millipore, Billerica, MA, USA). Briefly, a quantity of 40 mg liver tissue was incubated with 1% formaldehyde for 10 min at room temperature to cross link proteins and DNA. Cross-linking was terminated by the addition of glycine (1 M). The liver tissue was washed twice with cold PBS and placed in 1 ML of SDS lysis buffer containing 5 uL1X protease inhibitor cocktail II. The lysates were aliquoted to 300~400 uL per microfuge tube and sonicated on ice to produce sheared, soluble chromatin (−200~1000 base pairs). Added 900 uL of Dilution Buffer containing Protease Inhibitor Cocktail II into each tube containing 100 uL of sheared crosslinked chromatin. Each of the tubes was precleared with protein G Agarose (60 uL) at 4 °C for rotating 1 hour. The samples were microfuged at 4000 g for 1 minute to pellet agarose, and the supernatant was placed in new tubes with removing 10 uL as Input. The supernatant fractions were incubated with antibodies against acetylated acelated histone H3 (2 ul, catalog no. 17–10254, Millipore, Billerica, MA, USA) at 4 °C overnight. Protein G Agarose (60 uL) were added to each tube, the mixtures incubated for 1 h at 4 °C, and the immune complexes collected by centrifugation. The beads containing the immunoprecipitated complexes were washed sequentially for 5 min in Low Salt Immune Complex Wash Buffer, High Salt Immune Complex Wash Buffer, LiCI Immune Complex Wash Buffer, and in 2X TE buffer. The beads were eluted with 100 uL elution buffer (5 uL 20% SDS, 10 uL 1 M NaHCO_3_, 85 uL sterile distilled water) at room temperature. This was repeated once and eluates were combined. The input tubes were also added 200 uL of elution buffer. Cross-linking of the immuneprecipitated chromatin complexes and input controls was reversed by heating the samples at 65 °C for 5 h after adding 8 uL 5 M NaCl. Proteinase K was added to each sample in buffer (4 uL 0.5 M EDTA; 8 uL 1 M Tris-HCl) and incubated for 2 h at 45 °C.

Primers (A1: Forward: 5′-TAGGCTGCTTGAGCTGTGCG-3′, Reverse, 5′-ATAGTGCGGCGTGCTGTGAA-3′; A2: Forward: 5′-ATGTCATAAGCCAGAAGATGGG-3′, 5′-TTACTCGGTTCTAACGATAGGC-3′; A3: 5′-TAAAATGTCAGTTCATGGGGTGG-3′, 5′-AGGGGATTGATACAGTAGGGGGT-3′) around promoter were used to examine the binding of acetylation of histone H3 (K9, K14) at the promoter of AMPK-α1. For negative controls, we performed ChIP assays with an IgG antibody (1ug) followed by amplification of the promoter region (Forward: 5′-CGTAGCTCAGGCCTCTGCGCCCTT-3′, Reverse, 5′-CTGGCACTGCACAAGAAGATGCGGCTG-3′) of GAPDH to show immunospecificity of the antibodies for the AMPK-α1 promoter. The DNAs from the input, unbound, and bound fractions were determined by real-time quantitative PCR. Relative quantification of PCR products was based on value differences between the bound and input fractions using the ^ΔΔ^ *C*_t_ method.

### Statistical analysis

In our study, the experiment was repeated three times. Data were expressed as means ± SD. Comparisons of parameters between 2 groups were made by unpaired Student’s *t* test. One-way ANOVA followed by Bonferroni test was used for multiple comparisons between means. For clarity purposes, figures for Western blot shows 2 representative bands, depicting the highest and the lowest band intensity in each group. *P* < 0.05 and *P* < 0.01 were accepted as statistically significant.
